# Relation of inflammatory and oxidative biomarkers associated with relapse in pregnant women diagnosed with multiple sclerosis

**DOI:** 10.1590/1806-9282.20251979

**Published:** 2026-06-15

**Authors:** Burcu Bozkurt Özdal, Ayşe Gülçin Baştemur, İclal Sena Gezer, Burcu Kesikli, Ersin Kasım Ulusoy, Atakan Tanaçan, Nuray Yazıhan, Dilek Şahin

**Affiliations:** 1 Ankara Bilkent City Hospital, Department of Obstetrics and Gynecology, Division of Perinatology – Ankara, Turkey.; 2 Ankara University, Institute of Health Sciences, Department of Metabolism and Clinical Nutrition, Interdisciplinary Food – Ankara, Turkey.; 3 Ankara University, Faculty of Medicine, Department of Pathophysiology – Ankara, Turkey.; 4 Turkish Ministry of Health, Ankara City Hospital, Department of Neurology – Ankara, Turkey.

**Keywords:** Multiple sclerosis, Pregnancy, Interleukin-6, Interleukin-10, Tumor necrosis factor-alpha

## Abstract

**OBJECTIVE:**

The aim of this study was to examine the relationship between inflammatory mediators (tumor necrosis factor-alpha, interleukin-6, and interleukin-10), oxidative stress parameters, and obstetric-neonatal outcomes in maternal blood of pregnant patients with multiple sclerosis and healthy pregnant patients, and to identify biomarkers that may be useful in predicting relapse in pregnant patients with multiple sclerosis.

**METHODS:**

This prospective, single-center study was conducted at Ankara City Hospital between July 2024 and May 2025 and included a total of 72 patients: 36 pregnant women diagnosed with multiple sclerosis and 36 healthy pregnant women as the control group. The patients with multiple sclerosis were further divided into two groups: those who experienced relapses during pregnancy and those who did not. Clinical and demographic data, biochemical tests (interleukin-6, interleukin-10, tumor necrosis factor-alpha, total antioxidant status, and total oxidant status), and obstetric and neonatal outcomes were recorded. Data were analyzed statistically using SPSS 22.0, and appropriate statistical tests and correlation analyses were applied.

**RESULTS:**

Levels of interleukin-6, interleukin-10, tumor necrosis factor-alpha, total oxidant status, and total antioxidant status were significantly higher in patients with multiple sclerosis compared to controls (p<0.01). These parameters were even more elevated in patients who experienced relapses. Receiver operating characteristic analysis demonstrated that cut-off values of 237.8 pg/mL for tumor necrosis factor-alpha and 1.75 for interleukin-6/interleukin-10 provided high sensitivity and specificity in predicting multiple sclerosis relapse.

**CONCLUSION:**

Inflammatory and oxidative stress markers were elevated in pregnant women with multiple sclerosis, and tumor necrosis factor-alpha levels and the interleukin-6/interleukin-10 ratio were identified as potential biomarkers for predicting disease relapse.

## INTRODUCTION

Multiple sclerosis (MS) is a chronic, inflammatory, progressive degenerative disease of the central nervous system^1^. Although the onset of MS is most observed between the ages of 20 and 40, the female-to-male ratio in its incidence is 3:1^2^. Inflammation, demyelination, and axonal degeneration are the primary pathological mechanisms that lead to clinical manifestations^3^. However, the exact cause of MS remains unknown. The most widely accepted theory posits that T cells become autoreactive due to an unknown antigen, thereby initiating inflammation^4,5^. T cells are classified into T helper 1, which are proinflammatory, and T helper 2, which are anti-inflammatory. In the pathophysiology of MS, T helper 1 cells and the B cells they activate play a dominant role. T helper 1 cells exert their effects through cytokines such as interleukin (IL)-4, IL-5, IL-10, tumor necrosis factor-alpha (TNF-α), and tumor growth factor-beta. T cell activation leads to an increase in proinflammatory cytokines and oxidative stress, which in turn causes damage to the central nervous system^4^.

Oxidative stress plays a key role in MS pathogenesis by contributing to demyelination and neurodegeneration through an imbalance between reactive oxygen species (ROS) production and antioxidant defenses. This imbalance can be quantified using total oxidant status (TOS), which reflects overall oxidant levels, and total antioxidant status (TAS), which measures the cumulative capacity of antioxidants to neutralize ROS^6^. Decreased TAS and increased TOS have been observed in MS patients, correlating with disease severity and relapse activity^6^. During pregnancy, physiological adaptations such as hormonal shifts and immune modulation may alter this oxidative–antioxidative balance, potentially influencing MS relapse rates, though evidence in this specific context remains limited^7^.

During pregnancy, particularly in the third trimester, MS results in a 1-year delay in relapse occurrence. However, the relapse rate increases again during the puerperal period before returning to the pre-pregnancy rate^8^. There is a need for more evidence to determine the long-term effect of pregnancy on the progression of MS^9^.

The aim of this study was to examine the relationship between inflammatory mediators (TNF-α, IL-6, and IL-10), oxidative stress parameters, and obstetric-neonatal outcomes in the maternal blood of pregnant patients with MS and healthy pregnant patients and to identify biomarkers that may be useful in predicting relapse in pregnant patients with MS.

### Study population

This prospective, single-center study was conducted in a tertiary hospital. The study included patients followed up with a diagnosis of MS between July 2024 and May 2025 in the High-Risk Pregnancy Unit of Ankara City Hospital who were between the 28th and 41st weeks of gestation. Each patient included in the study signed an informed consent form. Ethical approval for the study was obtained from the Ethics Committee of Ankara City Hospital (TABED-1-24-91). All stages of the study were conducted in accordance with the principles of the Declaration of Helsinki.

Patients were followed up from the first presentation until delivery. We are one of the largest hospitals in Turkey, and we follow-up maternal diseases. Notably, four patients had an attack in the first two trimesters during pregnancy. Birth outcomes were recorded.

For each patient included in the study, the following clinical and demographic data were recorded: age, parity, gravida, duration since MS diagnosis, MS type, presence of MS relapse during pregnancy, expanded disability status scale (edss) score, gestational week at the time of maternal blood testing, maternal blood levels of neutrophils, neutrophil percentage, lymphocytes, lymphocyte percentage, basophils, basophil percentage, eosinophils, eosinophil percentage, IL-6, IL-10, TNF-α, TAS, total oxidative stress (TOS), estradiol, gestational age at delivery, neonatal birth weight, first- and fifth-minute Apgar scores, neonatal cord arterial pH, and whether the neonate was admitted to the neonatal intensive care unit (NICU).

Patients included in the control group were selected randomly from healthy pregnant women. For each patient in the case group, one control patient was included. Care was taken to ensure an equal distribution of gestational weeks between the case and control groups. Patients with multiple pregnancies, organ transplants, immunodeficiencies, autoimmune diseases, inflammatory diseases, or known major fetal chromosomal or cardiac structural anomalies, as well as those with incomplete or inaccessible data, were excluded from the study.

### Measurement of cytokine levels

The blood samples were collected and centrifuged for 5 min at 4°C at 3,000 rpm. Supernatants were used for evaluation of the proinflammatory and inflammatory cytokine levels, as well as the general oxidative and antioxidative status. UV–VIS spectrophotometer (Epoch; Biotech, USA) is used for all measurements. Cytokine levels (TNF-α, IL-6, and IL-10) were quantified and presented in pg/mL using commercial ELISA kits (e-Biosciences ELISA Ready SET-Go Affymetrix, Thermo Scientific)^10^.

### Analysis of oxidant/antioxidant levels

Using commercially available products (Rel Assay Kit Diagnostics, Turkey), a spectrophotometric technique was used to assess the total antioxidant and oxidant status. TAS was measured and presented in mmol Trolox Eq/L, calibrated using Trolox, a water-soluble homologue of vitamin E, while TOS was measured and presented in μmol H2O2 Eq/L, calibrated using hydrogen peroxide^10^.

### Statistical analysis

Statistical analyses were conducted using SPSS version 22.0 (SPSS Inc., Chicago, IL, USA). The Kolmogorov-Smirnov and Shapiro-Wilk tests were used to assess the normality of the data distribution. The Mann-Whitney U test was employed to compare non-normally distributed variables. Descriptive analyses were presented as median and minimum–maximum for non-normally distributed variables. The chi-square test was used to compare categorical variables. In addition, Spearman’s correlation coefficient was utilized to examine the relationship between variables and MS relapse. Receiver operating characteristic (ROC) curve analysis was used to determine the cutoff point for predicting MS relapse. A p-value of less than 0.05 was considered statistically significant.

## RESULTS

A total of 72 patients were included in this study, comprising 36 pregnant women diagnosed with MS and 36 healthy controls. The patients with MS were further divided into two groups based on the presence of relapse during pregnancy. Among these, 4 patients (11.1%) experienced a relapse during pregnancy, while 32 patients (88.9%) did not. edss system was used for the disease levels of MS patients. According to edss, 1 patient was scored 6 points, 1 patient was scored 3.5 points, and the other patients were scored between 0 and 2 points.

Only 1 of the MS patients had a diagnosis of progressive MS, while the remaining were diagnosed with relapsing-remitting MS (RRMS). All patients with MS had discontinued their medication during pregnancy. Among the four patients who experienced a relapse, three had their relapse in the first trimester and one in the second trimester.

The clinical-demographic data and biochemical results of the MS and control groups are presented in Table 1. Age, gestational week at the time of blood sampling, parity, abortion history, C-reactive protein, neutrophil count, neutrophil percentage, basophil count, basophil percentage, eosinophil count, eosinophil percentage, lymphocyte count, and lymphocyte percentage were found to be similar between the groups (p>0.05). However, statistically significant differences were observed between the groups in terms of the IL-6, IL-10, IL-6/IL-10, TNF-α, TAS, TOS, and TAS/TOS values (p<0.01). The two groups did not significantly differ in relation to gestational age at delivery, birth weight, first- and fifth-minute Apgar scores, or NICU admission (p>0.05).

**Table 1:** Demographic and biochemical data of pregnant women diagnosed with multiple sclerosis and healthy pregnant controls.

Variable	MS group n=36 Median (min–max)	Control group n=36 Median (min–max)	p-value
Age (years)	34 (25–38)	32 (21–41)	0.531
Gestational age (weeks)	32.0 (28.1–40.4)	31.3 (28.4–41.0)	0.893
Parity	1.0 (1.0–4.0)	2.0 (1.0–5.0)	0.370
Miscarriage	0.0 (0.0–2.0)	0.0 (0.0–2.0)	0.516
C-reactive protein	7.5 (1.2–121.3)	8.8 (0.0–60.6)	0.440
Neutrophil count	7.8 (0.0–12.9)	7.3 (3.5–68.5)	0.494
Neutrophil percentage	72.3 (0.40–89.8)	72.9 (2.5–88.5)	0.801
Basophil count	0.02 (0.01–0.06)	0.02 (0.01–0.20)	0.447
Basophil percentage	0.20 (0.10–0.50)	0.20 (0.10–9.5)	0.836
Eosinophil count	0.09 (0.01–0.38)	0.06 (0.01–3.90)	0.660
Eosinophil percentage	1.0 (0.10–5.60)	0.70 (0.02–10.0)	0.847
Lymphocyte count	1.70 (0.56–5.41)	1.85 (0.75–21.8)	0.380
Lymphocyte percentage	18.3 (5.70–30.9)	18.7 (0.53–32.8)	0.732
IL-6	120.1 (19.3–555.9)	27.4 (8.18–221.1)	**<0.001**
IL-10	81.7 (28.2–244.0)	126.3 (68.9–337.7)	**<0.001**
IL-6/IL-10	1.36 (0.27–5.89)	1.36 (0.27–5.89)	**<0.001**
TNF-α	189.7 (53.3–589.8)	81.8 (3.5–140.5)	**<0.001**
TAS	0.723 (0.154–0.978)	0.927 (0.745–1.031)	**<0.001**
TOS	18.73 (10.0–38.2)	10.92 (2.08–30.62)	**<0.001**
TAS/TOS	0.032 (0.008–0.073)	0.076 (0.45–1.16)	**<0.001**
Neonatal outcomes			
Gestational age (weeks)	38.0 (34.0–40.5)	37.6 (37.0–40.6)	0.660
Birth weight (g)	3,170 (2,160–4,315)	3,200 (3,000–4,220)	0.471
First-minute Apgar score	8.0 (6.0–9.0)	8.0 (5.0–9.0)	0.468
Fifth-minute Apgar score	9.0 (7.0–10.0)	9.0 (7.0–9.0)	0.486
NICU admission	0 (0%)	1 (2.7%)	0.292

MS: multiple sclerosis; NICU: neonatal intensive care unit; TNF-α: tumor necrosis factor-alpha; IL: interleukin; TAS: total antioxidant status; TOS: total oxidant status. TNF-α, IL-6, and IL-10 are presented in pg/mL, TAS: mmol Trolox Eq/L, TOS: μmol H_2_O_2_ Eq/L. Mann-Whitney U test was applied for parametric variables. p<0.05 is statistically significant and are presented in bold.

The patients with MS were further divided into two subgroups based on the presence or absence of relapse during pregnancy. The clinical-demographic and biochemical data of these subgroups are presented in Table 2. No statistically significant differences were found between these groups in terms of age, gravida, parity, neutrophil count, neutrophil percentage, basophil count, basophil percentage, eosinophil count, eosinophil percentage, lymphocyte percentage, IL-6, IL-10, TAS, TOS, or TAS/TOS (p>0.05). However, in the group that experienced relapse during pregnancy, duration since MS diagnosis, C-reactive protein, lymphocyte count, IL-6/IL-10, and TNF-α levels were significantly higher compared to the non-relapse group (p<0.05).

**Table 2:** Demographic and biochemical data of multiple sclerosis cases with and without relapse during pregnancy.

Variable	Relapse n=4 Median (min–max)	Non-relapse n=32 Median (min–max)	p-value
Age (years)	36 (25–38)	30.5 (28–33)	0.628
Gestational age (weeks)	32 (28–38)	31.4 (28–38.4)	0.745
Gravidity	2.0 (1.0–4.0)	1.5 (1.0–2.0)	0.574
Parity	1.0 (0.0–3.0)	1.0 (0.0–2.0)	0.436
C-reactive protein	14.3 (11.2–17.42)	8.94 (1.9–12.2)	**0.038**
Duration since MS diagnosis	8.0 (7.0–13.0)	5.0 (5.0–5.0)	**0.002**
Neutrophil count	6.51 (6.12–11.6)	7.74 (6.2–9.2)	0.270
Neutrophil percentage	66.1 (63.3–74.1)	78.8 (77.6–80.0)	0.255
Basophil count	0.02 (0.02–0.06)	0.01 (0.01–0.02)	0.898
Basophil percentage	0.20 (0.20–0.30)	0.10 (0.10–0.10)	0.705
Eosinophil count	0.07 (0.06–0.08)	0.03 (0.03–0.04)	0.557
Eosinophil percentage	0.70 (0.30–0.90)	0.40 (0.30–0.50)	0.426
Lymphocyte count	2.4 (1.5–5.4)	1.5 (1.0–2.0)	**0.024**
Lymphocyte percentage	24.8 (19.3–29.3)	15.4 (13.7–17.1)	0.116
IL-6	329.1 (133.6–368.6)	55.7 (50.3–61.1)	0.091
IL-10	102.1 (101.7–175.5)	58.5 (53.2–63.9)	0.079
IL-6/IL-10	2.25 (1.77–3.88)	0.60 (0.03–5.89)	**0.034**
TNF-α	238.9 (192.4–345.4)	146.5 (53.3–239.7)	**0.013**
TAS	0.72 (0.71–0.73)	0.81 (0.81–0.82)	0.119
TOS	36.6 (18.4–38.2)	25.1 (18.5–31.8)	0.422
TAS/TOS	0.019 (0.018–0.039)	0.034 (0.025–0.043)	0.258
Estriol	0.63 (0.49–0.70)	0.80 (0.58–1.02)	0.286

MS: multiple sclerosis, TNF-α: tumor necrosis factor-alpha, IL: interleukin, TAS: total antioxidant status, TOS: total oxidant status. TNF-α, IL-6, and IL-10 are presented in pg/mL, TAS: mmol Trolox Eq/L, TOS: μmol H_2_O_2_ Eq/L. Mann-Whitney U test was applied for parametric variables. p<0.05 is statistically significant and are presented in bold.

Among the variables that differed significantly between the relapse and non-relapse groups, namely, duration since MS diagnosis, TNF-α, IL-6/IL-10, and lymphocyte levels, Spearman correlation analysis revealed that TNF-α and MS diagnosis duration had a positive correlation with relapse occurrence (r=0.486, p<0.001).

**Figure 1:**
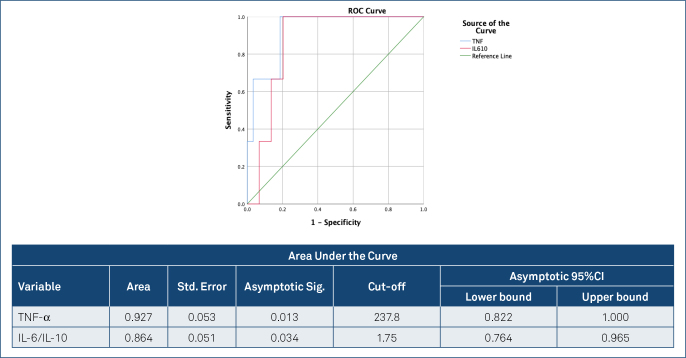
Receiver operating characteristic analysis of tumor necrosis factor-alpha and the interleukin-6/interleukin-10 ratio for predicting multiple sclerosis relapse.

Based on the ROC curve analysis, the optimal cut-off value of TNF-α for predicting MS relapse was determined to be 237.8, with 100% sensitivity and 91% specificity (area under the curve=0.927, p=0.013). The optimal cut-off value of the IL-6/IL-10 ratio for predicting MS relapse was 1.75, with 100% sensitivity and 80% specificity (area under the curve=0.864, p=0.034) (Figure 1).

Patients were grouped according to edss score, but no significant difference was detected in cytokines, TAS, and TOS values.

## DISCUSSION

In this study, we compared the proinflammatory and anti-inflammatory cytokines (TNF-α, IL-6, and IL-10) and oxidative stress mediators (TAS and TOS) between pregnant women diagnosed with MS and healthy pregnant controls. We determined that pregnant women with MS had elevated levels of proinflammatory cytokines (IL-6 and TNF-α) and reduced levels of anti-inflammatory cytokines (IL-10). Simultaneously, oxidative stress parameters were found to be increased, while antioxidant parameters were decreased. This suggests that the inflammatory processes involved in the pathophysiology of MS persist during pregnancy.

We also compared the same cytokines between patients with MS who experienced relapses during pregnancy and those who did not. We found that TNF-α and IL-6/IL-10 were significantly higher in those who experienced relapses. These biochemical values were shown to be positively correlated with MS relapse, and their cut-off values were calculated for predicting relapse.

MS is an autoimmune, chronic inflammatory disease. Although its exact etiology remains unclear, the pathophysiological mechanisms are believed to involve proinflammatory cytokines generated by T lymphocyte activation^11^. In the current study, we investigated several of these cytokines. Compared to healthy pregnant individuals, patients with MS demonstrated elevated levels of proinflammatory cytokines and reduced levels of anti-inflammatory cytokines.

Most MS cases have the RRMS subtype, which is characterized by new neurological symptoms or worsening of existing symptoms due to recurrent inflammatory attacks. A smaller proportion have progressive MS, where neurological findings progress independently of relapses. In our patient cohort, 35 cases were classified as RRMS and only 1 as progressive MS^12^.

Due to hormonal changes, the relapse rate in patients with MS tends to decrease, particularly in the second and third trimesters of pregnancy, and increases again within the first 3 months postpartum^1^. The POPART’MUS trial demonstrated a significant reduction in MS relapses during the third trimester of pregnancy and reported that high-dose progesterone administration postpartum reduced relapse incidence^13^. A study conducted across 12 European countries reported a relapse frequency of 10% in pregnant patients with MS^14^, which is similar to the relapse rate observed in our study.

To our knowledge, there are no previous studies in the literature examining cytokine levels in pregnant women diagnosed with MS. Existing studies on cytokines have been primarily conducted in non-pregnant MS populations. One study assessing IL-12 and TNF-α in the cerebrospinal fluid of patients with MS found elevated TNF-α levels in 60% of active MS cases and associated these levels with disability severity^15^. Treatments used in MS are generally discontinued in patients who are planning pregnancy^16^. All pregnant patients with MS who participated in the current study had ceased their medications. Among the four patients who experienced MS relapses during pregnancy, three had relapses in the first trimester and one in the second trimester. When comparing patients who experienced relapses with those in remission, TNF-α was identified as the most significantly elevated cytokine during relapse.

Another study comparing patients with MS to healthy individuals found significantly elevated serum levels of proinflammatory cytokines such as TNF-α and IL-1 in the former^17^. A comprehensive review reported an inverse correlation between TNF-α levels and cognitive function in patients with MS^18^. Yet another study identified increased TNF-α and decreased IL-10 levels in MS cases^19^.

There is a body of literature indicating that TNF-α exerts its effects through various mechanisms. Some studies have shown that TNF-α, and to a lesser extent IL-1β, exacerbate the neurodegenerative process through the activation of various enzymes^20^, while others suggest that inflammation-induced hypoxia leads to neurodegeneration^17^.

In addition to our findings on cytokine imbalances, the assessment of oxidative stress through TAS and TOS provides important insights into the pathophysiology of MS during pregnancy. Previous studies have shown that TAS levels decrease while TOS increases in chemically induced toxicity models, highlighting the protective role of antioxidants such as thymoquinone in restoring oxidative balance^21^. This is consistent with our observation that TOS is elevated and TAS is altered in MS patients, indicating persistent oxidative damage. Furthermore, in studies examining the effects of acrylamide on lung tissue, TAS and TOS parameters after crocin treatment were improved, highlighting the potential of therapeutic interventions to reduce oxidative stress in inflammatory conditions. These results are parallel to our data showing that TAS/TOS imbalances are more pronounced in relapsing MS cases^22^. Additional studies confirm that TAS and TOS function as reliable biomarkers for assessing oxidative–antioxidant dynamics in various pathological conditions and are useful for predicting MS relapse during pregnancy^23^. Integrating such biomarkers may improve clinical monitoring and management strategies for pregnant women with MS.

This study has several important limitations. The first is the relatively small sample size, which limits both the statistical power and generalizability of the results. However, the specificity of the study population, namely pregnant women diagnosed with MS, contributed to the limited sample size. The single-center design also means that the results may reflect regional and demographic characteristics and highlight the need for multicenter studies covering more diverse geographical areas. Lastly, only a limited number of cytokines (IL-6, IL-10, and TNF-α) and oxidative stress parameters (TAS and TOS) were evaluated in this study. Other potential biomarkers associated with inflammation and oxidative stress, such as IL-12, interferon-gamma, superoxide dismutase, and glutathione peroxidase, were not assessed.

One of the principal strengths of this study lies in its comprehensive assessment of clinical, biochemical, and obstetric parameters, which uniquely contributes to the literature. By providing a detailed analysis of the relationship between MS and inflammatory cytokines during pregnancy, this study stands out among the limited number of investigations in this area. It serves as a valuable reference for the potential clinical application of these findings in predicting MS relapses and informs future research and therapeutic strategies.

In conclusion, this preliminary study identified differences in proinflammatory cytokines (TNF-α and IL-6), anti-inflammatory cytokines (IL-10), and oxidative stress parameters (TAS and TOS) in pregnant women diagnosed with MS. Notably, those who experienced MS relapses showed elevated TNF-α levels and IL-6/IL-10 ratios, with these parameters exhibiting potential associations with relapse occurrence in our limited cohort. These initial findings suggest that inflammatory and oxidative stress mediators could be explored as candidate biomarkers in the context of MS during pregnancy. However, due to the small sample size, larger-scale, multicenter studies are essential to validate these observations, assess their clinical utility, and inform potential management strategies.

## Data Availability

The datasets generated and/or analyzed during the current study are available from the corresponding author upon reasonable request.
